# Alternative Sigma Factor σ^H^ Modulates Prophage Integration and Excision in *Staphylococcus aureus*

**DOI:** 10.1371/journal.ppat.1000888

**Published:** 2010-05-13

**Authors:** Liang Tao, Xiaoqian Wu, Baolin Sun

**Affiliations:** Hefei National Laboratory for Physical Sciences at Microscale and School of Life Sciences, University of Science and Technology of China, Hefei, Anhui, China; Dartmouth Medical School, United States of America

## Abstract

The prophage is one of the most important components of variable regions in bacterial genomes. Some prophages carry additional genes that may enhance the toxicity and survival ability of their host bacteria. This phenomenon is predominant in *Staphylococcus aureus*, a very common human pathogen. Bioinformatics analysis of several staphylococcal prophages revealed a highly conserved 40-bp untranslated region upstream of the *int* gene. A small transcript encoding phage integrase was identified to be initiated from the region, demonstrating that the untranslated region contained a promoter for *int*. No typical recognition sequence for either σ^A^ or σ^B^ was identified in the 40-bp region. Experiments both *in vitro* and *in vivo* demonstrated that σ^H^ recognized the promoter and directed transcription. Genetic deletion of *sigH* altered the *int* expression, and subsequently, the excision proportion of prophage DNAs. Phage assays further showed that sigH affected the ability of spontaneous lysis and lysogenization in *S. aureus*, suggesting that sigH plays a role in stabilizing the lysogenic state. These findings revealed a novel mechanism of prophage integration specifically regulated by a host-source alternative sigma factor. This mechanism suggests a co-evolution strategy of staphylococcal prophages and their host bacteria.

## Author Summary

*Staphylococcus aureus* is a widely distributed opportunistic pathogen causing numerous foreign-body-associated infections. A large group of virulence factors are encoded by genes of prophages integrated in the bacterial genome. Here we show a heretofore unrecognized mechanism whereby an alternative sigma factor is recruited by a staphylococcal temperate phage for the regulation of *int* transcription. The modulation is processed via a direct recognition of a newly defined *int* promoter, while the integrase has critical roles in prophage integration and excision. The recruitment of a host-source sigma factor for integration modulation may provide the prophage with a novel strategy to sense the host conditions and further influence prophage gene expression and correlative bacterial virulence.

## Introduction

Prophages are viral cellular parasites that integrate into bacterial genomes and co-replicate with host chromosomes. A subset of bacteriophage genomes encodes additional virulence factors, and the production of these virulence factors can enhance bacterial toxicity as well as survival ability in various environments [1], [2]. Prophages are not rare in the chromosome of *Staphylococcus aureus,* a widely spread human pathogen. In fact, most clinical isolates harbor at least one prophage [3]. Many staphylococcal phages contain genes that encode virulence factors such as staphylokinase, enterotoxin A, chemotaxis inhibitory protein, staphylococcal complement inhibitor and leukocidin, which greatly enhance the bacterial invasiveness and help to evade host immunity in organic infection [4], [5], [6]. The transfer of toxic genes by a lysogenic bacteriophage, or phage conversion, is an important mechanism in the evolution of virulent *S. aureus* strains [7]. Furthermore, phages participate in the mediation of horizontal transfer of pathogenicity islands and raise intra-strain and inter-strain exchange frequency of toxic genes [8], [9].

As a member of double-strand DNA viruses, a temperate phage needs to recruit bacterial RNA polymerase with essential sigma factors to initiate its cascade. In *S. aureus*, only four sigma factors have been identified to date: σ^A^, the housekeeping sigma factor, which directs the transcription of the bulk cellular RNA, and three alternative sigma factors σ^B^ [10], σ^H^ [11], and the newly defined σ^S^ [12]. The *S. aureus* σ^B^ protein is closely related to the σ^B^ protein of *Bacillus subtilis*, and is mainly involved in stress response [13], [14]. The *S. aureus* σ^H^ protein is a homolog of *B. subtilis* σ^H^, which regulates sporulation-related genes [15].

At the turn of the 21^st^ century, *S. aureus* genome nucleotide sequences were being completed at a rapid and increasing rate [16]. At least 14 *S. aureus* strains have been whole-genome sequenced and more are in progress. In addition, many staphylococcal phages have been identified independently and then sequenced as well [17], [18], [19], [20], [21], [22]. These sequenced genomes allowed us to comparatively analyze the staphylococcal prophage, one of the most important components of genomic variable regions and which provides numerous virulence factors to host bacteria. The majority of known staphylococcal bacteriophages belong to the order *Caudovirales* and the size of the phage genomes mainly ranges from 35 to 50 kb. Architectural analysis of these prophage genomes demonstrated identical gene arrangements (Figure S1). Some prophages share similar open reading frames (ORFs), especially in genes that encode products for the lytic cycle.

The integrase genes of these bacteriophages are highly conserved [23]. Some staphylococcal prophages even harbor the same integrase and insert into the same locus on the *S. aureus* genome. Besides integrase, the excisionase represents divergence among species. Indeed, only some of the staphylococcal prophages contain the *xis* gene, which encodes excisionase. The rest have a genetic structure called ORF-C, in the opposite direction of *int* [24]. Here we report a heretofore unrecognized manner whereby an alternative sigma factor is recruited by a staphylococcal temperate phage for the regulation of *int* transcription. The recognition site for σ^H^ is upstream of the ORF of *int*, which encodes phage integrase. Deletion of *sigH* resulted in a decrease of phage *int* mRNA level and an increase of the excised form of prophage genomes under normal growth conditions. The abilities of spontaneous lysis and lysogenization in *S. aureus* were also affected. These results indicate that *S. aureus* σ^H^ modulates the transcription of phage integrase, stabilizes the lysogeny in the host cell and may further influence the prophage life cycle and correlative bacterial virulence.

## Results

### Comparative analysis of *S. aureus* prophage genomes identified a highly conserved region upstream of the *int* gene

**Figure 1:**
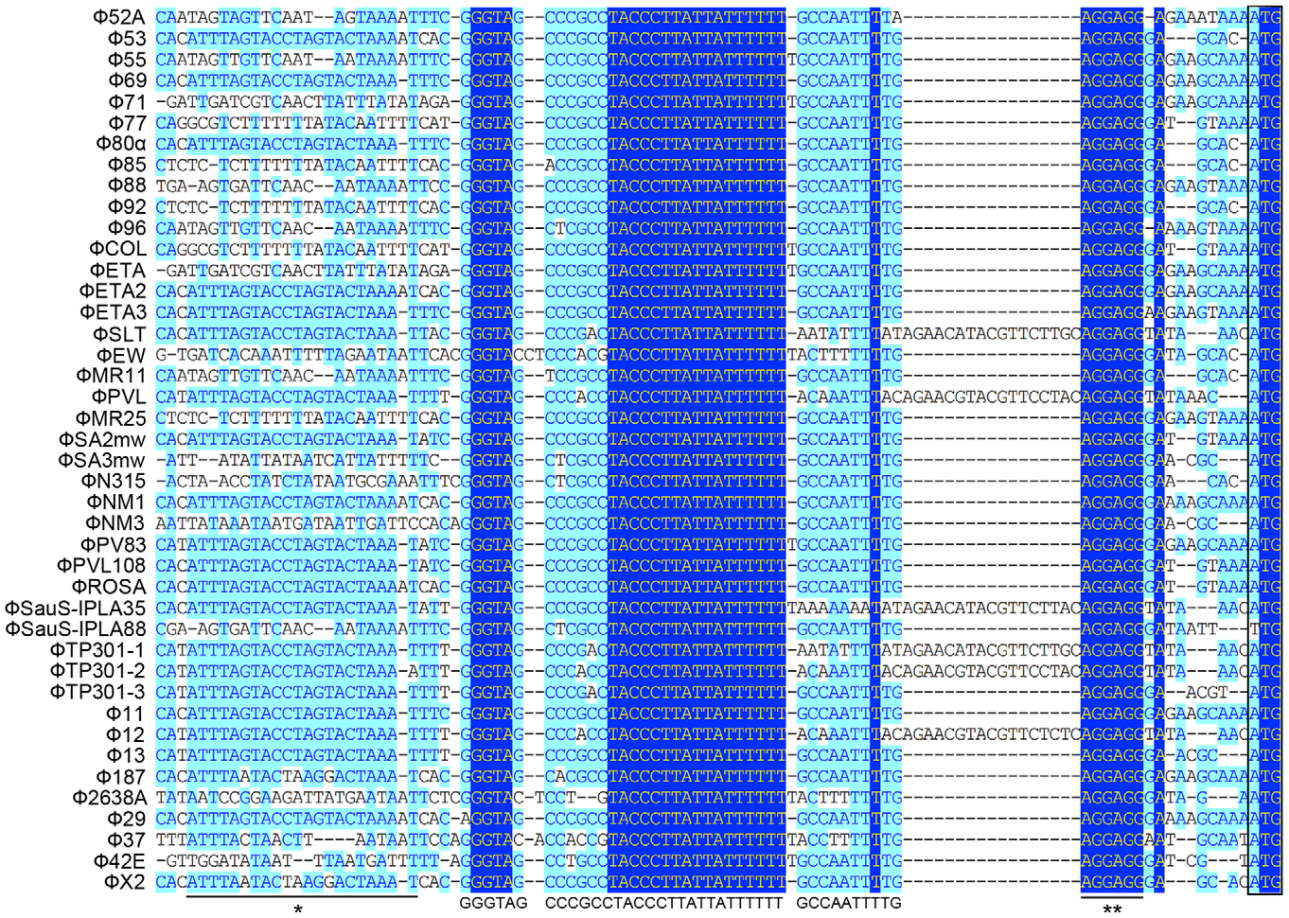
Sequence analysis of 5′-UTR of *int* in 42 *S. aureus* prophages. A highly conserved region (GGGTAGCCCGCCTACCCTTATTATTTTTTGCCAATTT) is identified. The previous reported stem-loop structure (*) and SD sequence (**) are located at the two sides of the newly defined region. Light blue boxes indicate the conservative nucleotide bases and dark blue boxes indicate the identical bases among all sequences compared. The start codons of *int* genes are boxed.

We first analyzed 43 staphylococcal prophage sequences available from NCBI GenBank by multiple sequence alignment in segments. From the results of the *in silico* analysis we found that a small fragment in the 5′ untranslated region (UTR) of *int* was extremely conserved among nearly all the prophages that were compared (Figure 1), with the only exception of Φ3A, which is *int* defective [3]. The region was previous mentioned as junction A when compared six *S. aureus* phages [25]. The conserved region was a 40-bp fragment followed by the typical Shine-Dalgarno (SD) sequence AGGAGG and was closely related to *int*. More interestingly, a previously reported stem-loop structure of 9-base inverted repeat with a 3-base loop (ATTTAGTACtagGTACTAAAT) found in several staphylococcal prophages [24] was also adjacent to that region. These data indicated that the region was likely to be a transcriptional regulator binding domain.

**Figure 2:**
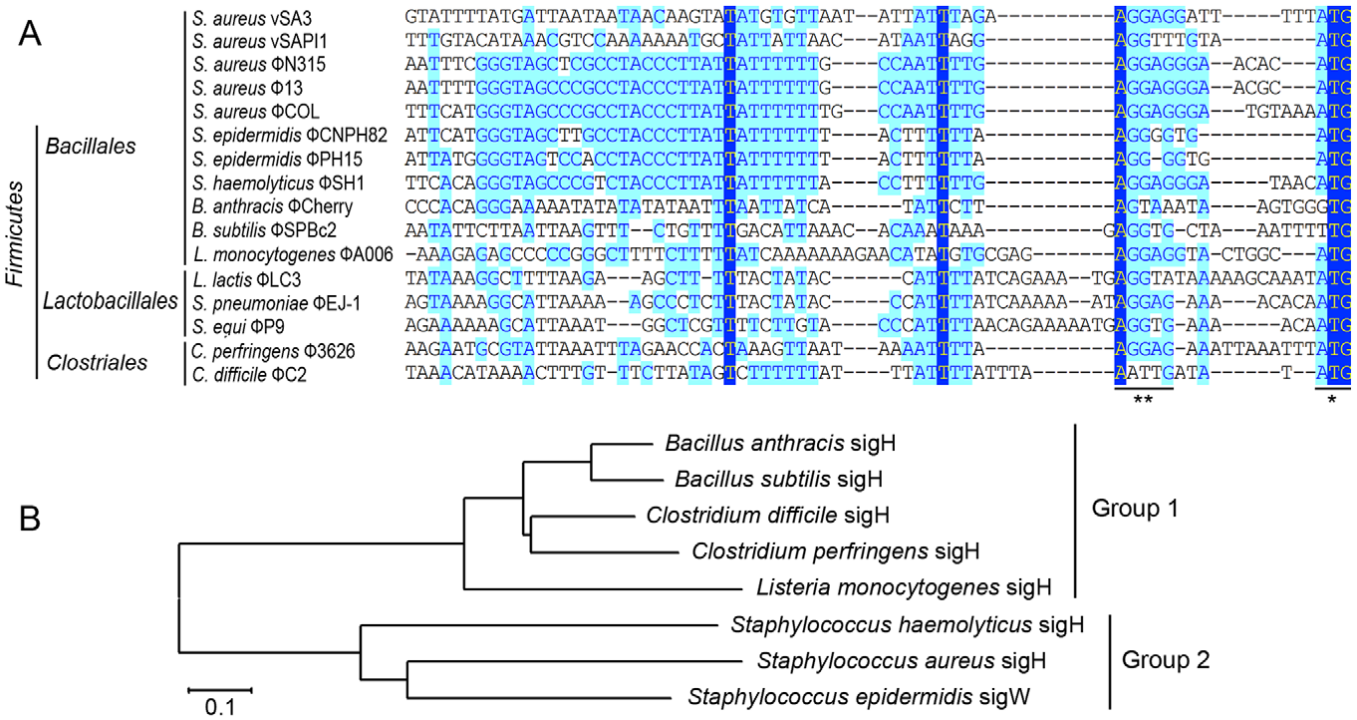
Sequence analysis of *int* promoters in several firmicutes harboring phages and a phylogenetic tree of sigH proteins in firmicutes. (A) A comparison of the DNA sequences upstream of *int* gene from several other firmicutes harboring prophages and two *S. aureus* pathogenic islands showing that similar sequences were also identified in *S. epidermidis* prophage ФCNPH82, ФPH15, and *S. haemolyticus* ФSH1. SD sequences and the start codons of *int* genes are indicated by asterisks. (B) A phylogenetic tree of sigH orthologs in the above firmicutes showed that staphylococcal sigH proteins were separated from the others by a deep node. Protein sequences were aligned using ClustalW in Vector NTI software. Alignments were imported into Mega 4 and the tree was generated using the neighbor-joining method, ignoring positions with gaps. The scale bar represents 0.1 substitutions per nucleotide site.

The DNA sequences upstream of *int* gene from several other firmicutes harboring prophages and two *S. aureus* pathogenic islands were also compared (Figure 2A). Similar sequences were identified in *Staphylococcus epidermidis* prophage ФCNPH82, ФPH15, and *Staphylococcus haemolyticus* ФSH1, but not in other prophages. It seemed that the conserved region only existed in the staphylococci harboring prophages.

### Analysis of the transcriptional organization of the *S. aureus* prophage *int* genes

**Figure 3:**
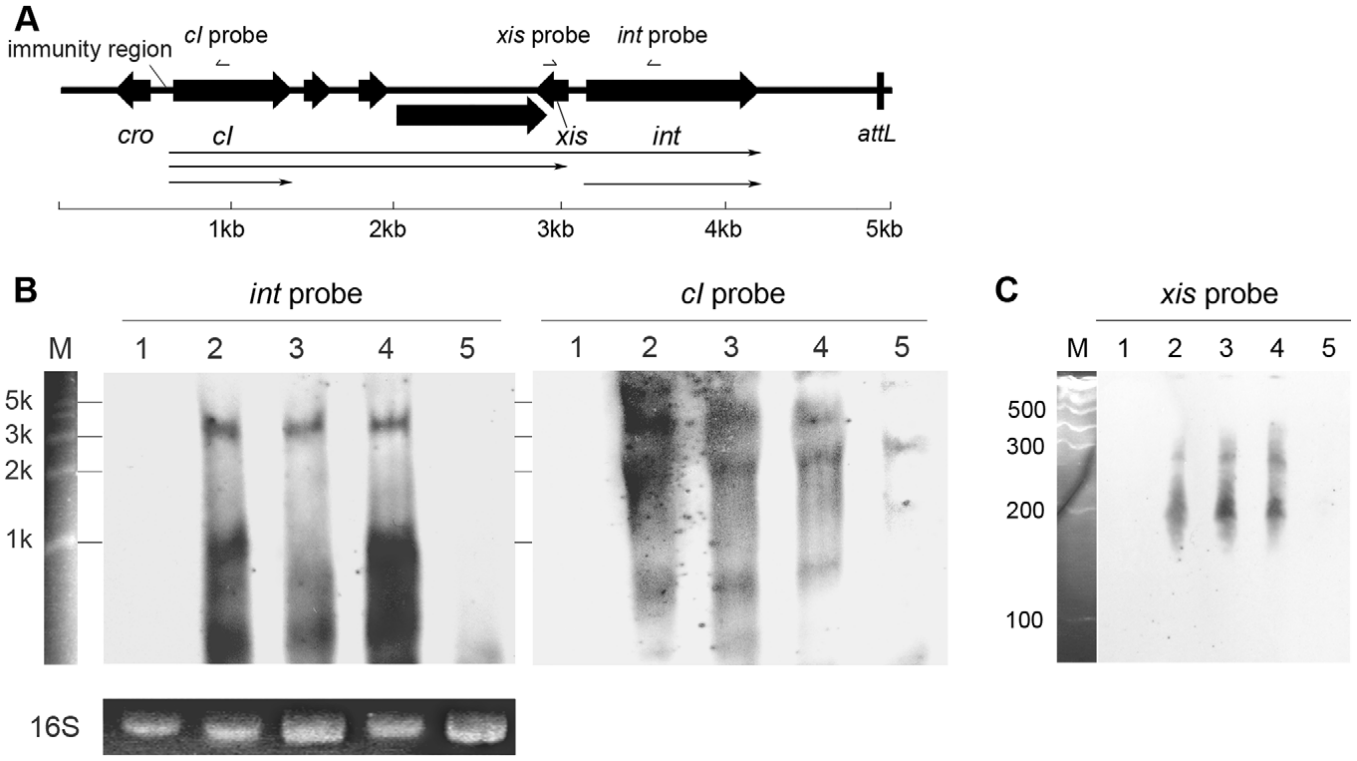
Transcriptional analysis of *int*, *cI*, and *xis* in Φ11. (A) Physical map of the gene organization of the left end of Ф11. The relative positions of the probes used in the Northern blot shown in panel B and C are indicated. The scale indicates the lengths of the genes. (B) Northern blots containing 5 µg of total RNA per lane were probed with Φ11 *int* probe (left) and reprobed with *cI* probe (right). Lane 1: phage cured strain RN4220; lane 2: Φ11 lysogen RN4220Φ11; lane 3: *sigH* mutant strain RN4220ΔsigHΦ11; lane 4: complementary strain RN4220ΔsigHcΦ11; lane 5: RN4220Φ11Δint. Ethidium bromide stained 16S rRNA patterns are shown as an indication of RNA loading. RL6000 (Takara) RNA marker (lane M) was used to estimate the molecular weight of the fragments. (C) Same RNA samples were probed with Φ11 *xis* probe at low molecular size. Lane 1: RN4220; lane 2: RN4220Φ11; lane 3: RN4220ΔsigHΦ11; lane 4: RN4220ΔsigHcΦ11; lane 5: RN4220Φ11Δint. RL1000 (Takara) RNA marker (lane M) was used to estimate the molecular weight of the fragments.

Since the results from *in silico* analysis strongly suggested that the newly defined region was a transcriptional regulation domain for *S. aureus* prophage *int* gene, it would be interesting to reveal the transcriptional organization of the integrase. Northern blot assays were performed to determine the transcriptional products for integrases in Ф11, Ф12, and Ф13, respectively. Under the normal lysogenic conditions, two mRNAs encoding phage integrase were produced. The larger mRNAs (∼3-kb to ∼4-kb) were initiated from promoters in the phage immunity region according to their lengths, and the smaller transcripts (∼1-kb) were presumably started from our newly defined region (Figures 3B and S2). To identify the exact transcriptional initiation site, a primer extension assay was carried out. The signal showed that the start site was just between the conserved region and the translational start codon (Figure S3). The results confirmed our hypothesis that the conserved region harbored a promoter specific for *int* expression. However, no typical sequences could be predicted as recognition motifs for neither σ^A^ nor stress response sigma factor σ^B^ in the newly defined region. Therefore, another protein needs to be recruited to function as a sigma factor. The protein could be either a phage gene product or another bacterial sigma factor.

### *S. aureus* sigma factor σ^H^ recognized the promoter for *int* and regulated its transcription

Therefore, we sought to identify this sigma factor in host bacteria. The alternative sigma factor σ^H^ was conserved in firmicutes and is known for its sporulation regulation function. Interestingly, staphylococci also express sigH but do not form spores. SigH proteins from staphylococci and other firmicutes were divided into two subgroups according to a previous report [11]. The phylogenetic analysis on sigH orthologs from several firmicutes showed that staphylococcal sigH proteins were separated from the others by a deep node (Figure 2B).

**Figure 4:**
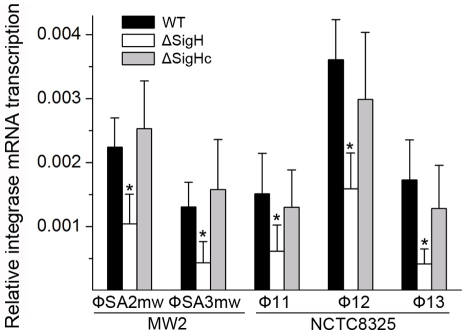
Alternative sigma factor σ^H^ up-regulates the mRNA levels of prophage integrases. Deletion of *sigH* causes a reduction of prophage integrase mRNAs. The complementation of *sigH* gene (ΔsigHc) by transforming pMAD*sigH* that integrated into host genome recovered the transcriptional levels of integrases. The integrases levels are indicated as the relative mRNA levels compared with the 16S RNA control (n = 3 samples/group, *p<0.05).

Following this, the *sigH* gene in *S. aureus* MW2 [26] genome was knocked out to generate ΔsigH (SUN0802) and a decrease in *int* mRNA levels of both ΦSa2mw and ΦSa3mw was observed by real-time quantitative reverse transcription polymerase chain reaction (Q-RT-PCR). To determine if this phenomenon was strain-specific, another *sigH* deletion strain (SUN0806) was built up in *S. aureus* NCTC8325 [27]. *Int* mRNA levels of Φ11, Φ12, and Φ13 were all reduced compared with the wild type (WT) (Figure 4). The *sigH* gene in RN4220 was also knocked out to generate strain SUN0914 for later experiments. To obtain the complementary strains (ΔsigHc), plasmid pMAD*sigH* was introduced into the above *sigH*-deficient strains and integrated into the host genomes; the backbone of the plasmid pMAD was then eliminated under the screening at 42°C. Endogenous *sigH* mRNA could be detected by RT-PCR in both WT and ΔsigHc but not in ΔsigH (Figure S4). As expected, *int* mRNA levels in ΔsigHc were recovered compared with ΔsigH (Figure 4). In the Northern blot assay, the absence of an mRNA signal of about 1-kb for Φ11 integrase was distinguished in ΔsigH compared with the WT and ΔsigHc (Figure 3B). Similarly, the hybridizing signals near 1-kb for Φ12 and Φ13 integrases were not detected in ΔsigH (Figure S2). The transcriptions of the ORF-C/*xis* gene on the opposite direction seemed to be unaffected without σ^H^ according to our Northern blots (Figures 3C and S2). However, when the gene locus of *int* was deleted, transcripts for ORF-C/*xis* gene could hardly be detected (Figure 3C), and the transcriptional level of *cI* also decreased (Figure 3B). These results demonstrated that the *S. aureus* alternative sigma factor σ^H^ plays a role in up-regulating the mRNA levels of prophage integrases. It was also interesting to note that the expression of sigH was growth-phase related (Figure S5).

**Figure 5:**
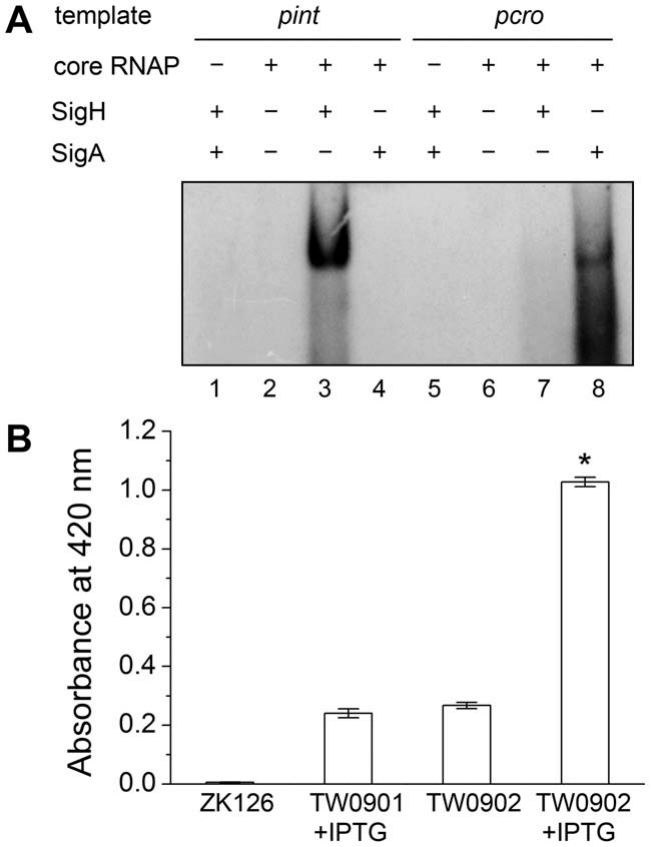
Direct evidence of σ^H^-dependent transcription from *pint* is observed both *in vitro* and *in vivo*. (A) *In vitro* transcription assay was performed using *E. coli* core RNA polymerase with or without σ^H^ or σ^A^. The absence or presence of core RNAP, σ^H^ and σ^A^ are indicated. Ten µCi of [α-^32^P]UTP was used in each sample for radioautography. (B) A *pint-lacZ* fusion was deployed for the determination of σ^H^-*pint* recognition *in vivo*. *LacZ*-deficient *E. coli* strain ZK126 was adopted as negative control. Fragment of *pint-lacZ* was introduced into ZK126 to generate TW0901. Strain TW0902 is derived from TW0901 by adding an IPTG-inducible plasmid pET22b*tac-sigH* that expresses *S. aureus* σ^H^. High activity of β-galactosidase was only detected in the group with *pint-lacZ* fusion and expressed *S. aureus* σ^H^ protein (n = 4 samples/group, *p<0.05 versus the controls).

To verify the direct interaction between σ^H^ and *int* promoter region, we conducted σ^H^-directed *in vitro* transcription by using an amplified DNA fragment from Φ11, which contained the conserved 40-bp region and part of the *int* ORF as a template. The core RNA polymerase was pre-incubated with σ^H^ and then incubated with the linear DNA template. Transcription was initiated by the addition of an NTP mixture containing [α-^32^P]UTP at 37°C. The σ^A^-dependent promoter of *cro* (*pcro*), an important promoter for transcription in the early period of the phage lytic cycle [28], and the *S. aureus* σ^A^ protein were chosen as controls. Core RNA polymerase pre-incubated with σ^H^ generated the expected signal at the presence of *pint,* while no corresponding signals were produced by core RNA polymerase, σ^A^-holoenzyme, or sigma factors alone (Figure 5A). By comparison of the intensity of the signals it was also found that the transcription from this σ^H^-dependent promoter was efficient *in vitro*. To investigate whether σ^H^-holoenzyme could recognize *pint* and produce transcripts *in vivo,* a conditional replication, integration, and modular vector pAH125 [29] was used to detect the recognition of the *pint* with the presence of σ^H^ in an *Escherichia coli* model. A small DNA fragment containing *pint* was inserted into pAH125 to build *pint*-*lacZ* fusion. The constructed plasmid pAH125*pint* was then transferred into *E. coli* strain ZK126 [30] and integrated into the bacterial genome at the *attP* site of the lambda phage with the help of pINT^ts^ [31] to obtain TW0901. Strain TW0902 was later obtained by transferring the plasmid pET22b*tac-sigH* that expresses *S. aureus* σ^H^ protein into TW0901 via electroporation. High activity of β-galactosidase was only exhibited in TW0902 after isopropyl β-D-1-thiogalactopyranoside (IPTG) induction (Figure 5B), confirming that *S. aureus* σ^H^ was specific for the *int* promoter recognition and gene transcription *in vivo*.

### The deletion of *sigH* caused an alteration in the proportion of the excised form of the prophage in *S. aureus*

**Figure 6:**
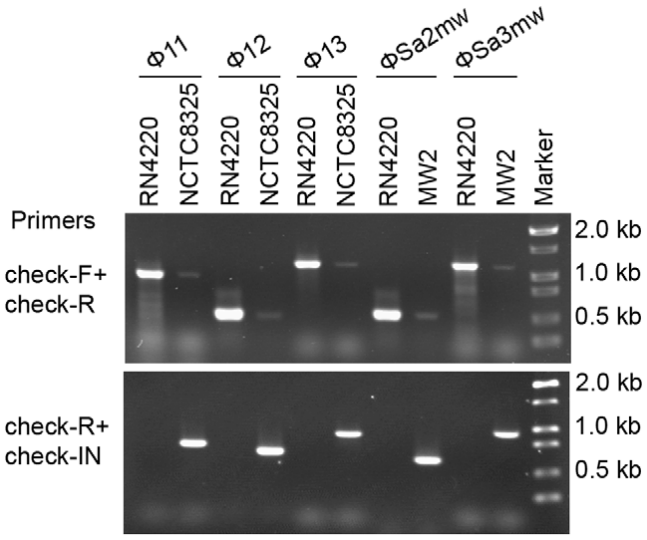
Staphylococcal prophages are mobile on host genomes by dynamic equilibrium of excision and reintegration. The excision of prophage from host genome is detected by PCR with primers check-Fs and check-Rs from two sides of the *attB* sites. Primers check-Rs outside the *attB* sites and check-INs inside the prophages, facing outwards, were used to detect the integrated prophage genomes. Phage-cured strain RN4220 was used as control.

Two forms of prophages naturally exist in many lysogenic bacteria, mostly in the integrated form and rarely in the excised form [32], [33], [34], [35], [36]. In *S. aureus*, we also observed the coexistence of both integrated and excised prophage DNAs by using a set of specially designed PCR primer pairs (Figure 6). Primers check-F and check-R were used to amplify the *attB* sites in *S. aureus* genome only if the prophages were excised. Primers check-R and check-IN were used to detect the presence of integrated phage genomes. In addition, by using a real-time Q-PCR method with the above primer pairs the excision frequencies of the staphylococcal prophages were estimated.

**Figure 7:**
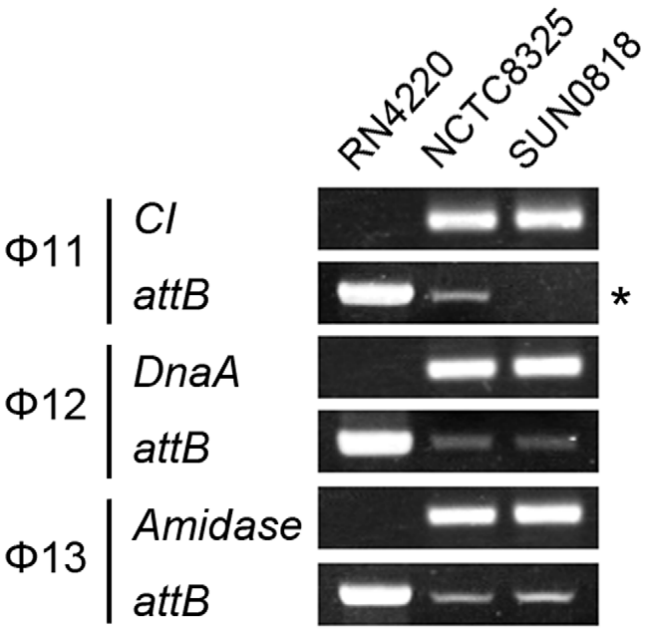
*Int* is essential for the process of excison. *CI* (Φ11), *dnaA* (Φ12), and *amidase* (Φ13) are phage genes that were used as endogenous controls. The deletion of Φ11 *int* makes prophage Φ11 immovable on its host genome (indicated by an asterisk) though integrases of Φ12 and Φ13 remain the same. Phage-cured strain RN4220 was used for positive control.

The *int* gene is required for both integration and excision of temperate phages [37] and some pathogenicity islands [38] as previously reported. We have verified the dual functions of *int* in the *S. aureus* prophages by constructing *int* deletion and overexpression strains. The Φ11 *int* mutant in *S. aureus* NCTC8325 (SUN0818) was generated to examine the excision of the prophage. No PCR products were observed using SUN0818 genome as templates with primer pair check11-F and check11-R (Figure 7), and the excision event could be recovered by introducing a complementary plasmid into the mutant strain (Figure S6). The Φ11 *int* overexpression strain was obtained by introducing PLI50Ф11*int* into RN4220Ф11. The Φ11 excision frequency in the overexpression strain was decreased and a lower titer was detected in the supernatant of the culture as expected (Figure S7), indicating that a higher *int* mRNA level had a role in stabilizing the lysogeny in the host genome.

**Figure 8:**
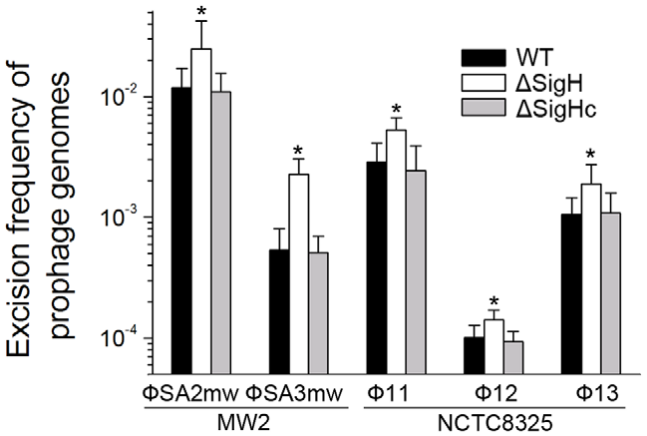
*SigH* plays a regulatory role on prophage excision and integration via integrase. Primer pairs from two sides of *attB* (check-F and check-R) were used for determination of the ratio of the excised phage genome. Primer pair with one primer outside the *att* site and the other primer inside the prophage (check-R and check-IN) was used for determination of the ratio of the integrated prophage. *SigH* deletion resulted in an increase in excised phage genomes. The ratios of excised prophage genomes were similar in WT and the complementation (n = 4 samples/group, *p<0.05).

Since sigH directly modulates the expression of phage integrase, we suspected that the defection of *sigH* gene would lead to a change in the number of excised circular phage DNAs. To test this, the proportion of excised prophage genomes in *sigH* mutant strains was compared to the WT. The proportions of the excised forms of the genomes of all five prophages tested, including ΦSa2mw, ΦSa3mw in *S. aureus* MW2 and Φ11, Φ12 and Φ13 in *S. aureus* NCTC8325, were significantly increased. In addition, the prophages excision frequencies were similar in WT and the complementation (Figure 8).

### Deletion of *sigH* altered the rate of spontaneous lysis and lysogenization in *S. aureus*

**Figure 9:**
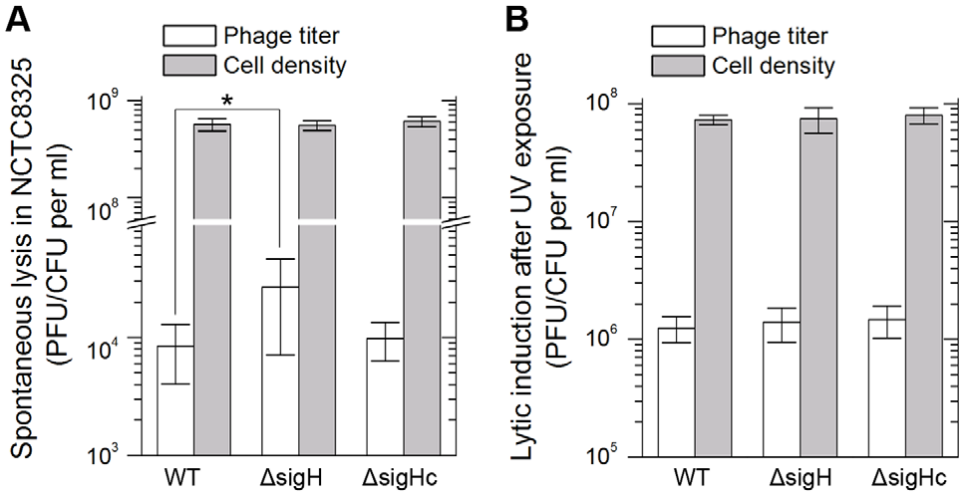
*SigH* deletion leads to an increase in spontaneous lysis. The frequencies of the spontaneous lysis of the lysogens were evaluated by determination of the phage titers of the culture supernatants. Susceptible strain RN4220 was used as the indicator. (A) The titer in the supernatant of the *sigH* mutant culture was much higher. (B) No difference of the titers among WT, the *sigH* mutant, and the complement was observed when the cells were pretreated with UV radiation for phage induction.

Integration and excision events are usually associated with the life cycle of the temperate phages [39]. We estimated the spontaneous lysis rate by determining the titer of free viral particles in *S. aureus* NCTC8325 culture at the exponential phase. The supernatants of the respective cultures were incubated with the indicator for adsorption and then plated onto agar plates to form plaques. A higher titer was detected in *sigH* mutant, suggesting that it was easier to naturally induce the prophages to a lytic cycle in the absence of sigH (Figure 9A). Besides, if the cultures were pretreated with ultraviolet (UV) radiation for phage induction, no differences of the titers could be observed between the supernatant of WT, *sigH* mutant and the complement cultures (Figure 9B). These results implied that sigH did not participate in the phage lytic cycle control.

We further investigated the effect of *sigH* deletion on the lysogenization ability of *S. aureus*. The susceptible strains RN4220, RN4220ΔsigH (SUN0914), and RN4220ΔSigHc were adsorbed by Ф11 particles with the same multiplicity and plated onto agar plates. The survival clones were checked for lysogeny by cross-streak assays. Fewer lysogens were formed in SUN0914, suggesting that the presence of sigH promoted the lysogenization in *S. aureus* (Table 1). These results demonstrated that sigH has an auxiliary role in maintaining the lysogeny in *S. aureus* by up-regulating the *int* mRNA level.

**Table 1:** Frequencies of lysogenization of *S. aureus* by Ф11 at the multiplicity of about 100.

*S. aureus*	Frequency of lysogenization	Standard deviation
RN4220	2.25×10^−2^	8.45×10^−3^
RN4220ΔsigH	4.49×10^−3^	1.71×10^−3^
RN4220ΔsigHc	1.79×10^−2^	6.65×10^−3^

## Discussion

Prophages, or lysogenic temperate viruses, provide a large group of virulence factors in bacterial pathogens. In addition, they facilitate the spread of phage genome-encoded or host genome-encoded virulence factors in different bacterial strains or even species [8]. Although the importance of prophage-related virulence in pathogenicity has gradually been realized [40], much is still unknown about how these virulence factors are modulated. The genomes of lysogenic phages do not permanently stay in its host genome. Occasionally, prophage genomes can be excised from bacterial chromosomes and integrated back later [37]. The mobility of a prophage genome usually reflects the activity of the phage although the detailed mechanism remains obscure. These reversible processes are commonly driven by the *int-xis* system in temperate phages, and some cofactors may participate as well [41].

The *int gene* is required for the process of integration, while both *int* and *xis* are required for the process of excision [37]. Structural analysis of the integrase family showed that their catalytic boxes are conserved. The major differences in the structures among the members of the integrase family mainly affect the specificity and efficiency of the reactions [42]. Although integrase has dual functions of both integration and excision, a higher *int* mRNA level usually promotes the reaction of integration that is required for lysogeny. The efficiency of the excision process mainly depends on the protein level of excisionase [39].

A similar regulation role was observed in *S. aureus* prophages. Although the *xis* genes are absent in some staphylococcal prophages, a gene structure called ORF-C may play a similar role instead [43]. The deletion of the gene locus of *int* in Ф11 resulted in a permanent lock of the prophage genome onto the host chromosome but not in a loss of the phage, while the excision and integration of Φ12 and Φ13 were not affected. No excision of the Ф11 genome could be detected though integrases of Φ12 and Φ13 still remained. The result reflected the high specificity of the integrases. The frequencies of the spontaneous excisions of prophage genomes varied among different *S. aureus* prophages. The ratio might partly depend on the sequence of attachment site, although some other factors are involved. For instance, the proportion of the naturally excised genome of ФSa2mw is 100 times of that of Ф12 even though they share the same *attB* site and integrase.

We uncovered the fact that the host alternative sigma factor σ^H^ directly modulated the transcription of *S. aureus* prophage integrase. The deletion of *sigH* caused an obvious decrease in integrase expression. This finding was confirmed by experiments both *in vitro* and *in vivo.* σ^H^ was also found to control the expression of the *comG* and *comE* operons, which may play roles in genetic competence in *S. aureus* [11]. Genetic competence may be defined as a physiological state for taking up high-molecular weight exogenous DNA [44]. But currently there has been no evidence that supports any connections between the phage's life cycle and the competent state of the host. While σ^A^ is commonly utilized for promoter recognition at the immunity region, the finding of the recruitment of a second sigma factor by the phage appears especially appealing. Besides, the Northern blot assay showed that integrase could be expressed by another long transcript from a σ^A^-dependent promoter. This result demonstrated that σ^H^ presumably affected the transcriptional level of integrase in a mildly regulative manner.

The deletion of *sigH* would cause an increase in naturally excised phage genomes. Because the process of integration and excision is reversible, the excised forms of phage genomes may reintegrate into the host genome or occasionally enter the lytic cycle. Studies on *S. aureus* prophage gene arrangement showed that *int* genes were adjacent to the *attL* site and usually no ORFs were found downstream in the same direction. And Northern blot assays showed that the transcription on the opposite direction was unaffected in the *sigH* mutant. Therefore, we deduced that the expression of neighboring genes (i.e. *xis*/ORF-C) should not be modulated by the *sigH* gene. It can be postulated that the increase in spontaneous phage excision was more likely a result of a decrease in reintegration rather than an increase in excision due to the reduction of integrase. It was previously reported that lysis genes were more active in excised phage DNA molecules in streptococcal prophages [34]. Here we also found that the expression of *cI* was decreased in the *int* mutant probably because of the immobility of the prophage. Besides, the spontaneous lysis rate in the *sigH* mutant was higher. This phenomenon was probably a consequence of a higher excision frequency of free circular phage genomes. Notably, the transcriptional levels of accessory virulence genes (i.e. *sak, sea*) were intimately associated with the phage's life cycle as well [28].

A lower rate of lysogenization was observed in the *sigH* mutant, probably due to the absence of the short transcript. Although the mRNA transcribed from a σ^A^-dependent promoter at the immunity region also expresses integrase, it is postulated that integrase expressed from the short transcript is dominant once a phage infects a bacterial cell because the σ^H^-dependent promoter is much closer to the ORF of *int*. This mechanism is very similar to the one in the well-studied bacteriophage lambda. A small transcript of *int* is produced from *p_RE_* under the stimulation of protein CІІ, while a long transcript from *p_L_* can also express integrase in a stable lysogenic state [45], [46]. Nevertheless, the difference between them is obvious in that CII protein is encoded by a phage gene at early stage to ensure the precise regulation [47] while sigH is a host-source protein. Although the absence of sigH does not cause an intense variation in the prophage characteristics, the presence of sigH has a distinct function in promoting and stabilizing the lysogeny of prophages in *S. aureus*. This may also explain why the conserved promoter region has been kept in most staphylococcal prophages during evolution but has never been found in other firmicutes harboring phages.

The regulation of sigH remains largely unknown. As an alternative sigma factor, the expression level of sigH would possibly respond to certain cellular conditions and/or variations in the environment. The findings suggest that the recruitment of sigH may provide the staphylococcal temperate phages with an additional strategy to sense the host conditions and/or the change of living environments.

This study could be vital in understanding the temperate phage lysogenic strategy and phage-related virulence. Further investigations would mainly concentrate on which host physiological conditions and/or their circumstance *S. aureus* sigH would respond to and how that process is regulated.

## Materials and Methods

### Strains, plasmids, and culture conditions

The bacterial strains, plasmids, and oligonucleotides used in this study are listed in Tables S1, S2, and S3. Bacteria were routinely grown in Luria-Bertani (LB) medium (for *E. coli*) or Tryptone Soy Broth (TSB, Oxiod) medium (for *S. aureus*) with aeration (200 rpm) at 37°C. For the antibiotics supplement in cultivation, 100 µg/ml ampicillin, 50 µg/ml kanamycin or 34 µg/ml chloromycetin was used for *E. coli* strains; 15 µg/ml chloromycetin or 10 µg/ml erythromycin was used for *S. aureus* strains. All plasmids used in *S. aureus* were first transferred into strain RN4220 [48] and then transformed into MW2 or NCTC8325 by electroporation.

### Genomic DNA extraction, RNA extraction, reverse transcription, quantitative PCR, and Northern blot assay

For genomic DNA or RNA extraction, one colony of each sample was inoculated in 5 ml of TSB medium and incubated at 37°C overnight. Each culture was started by diluting the precultures to an OD_600_ = 0.05 and was then incubated at 37°C (200 rpm). The cultivation was stopped at early exponential phase (OD_600_ = 0.2), mid-exponential phase (OD_600_ = 0.6), late-exponential phase (OD_600_ = 2.4), and stationary phase (OD_600_ = 4.0), respectively. *S. aureus* cells were pre-digested with digestion buffer containing 40 U/ml lysostaphin, 10 mg/ml lysozyme and 10% (v/v) glycerol. Genomic DNA was extracted using the EZ-10 Spin Column Genomic DNA Isolation Kit (Bio Basic Inc.). RNA extraction was performed using the SV Total RNA Isolation System (Promega). Residue DNA in extracted RNA was removed by treatment with 10 U of DNaseІ (Takara) at 37°C for 1 hour. RNA was purified by phenol-chloroform extraction and ethanol precipitation. Purified total RNA and genomic DNA were qualified and quantified by DU730 Nucleic Acid/Protein Analyzer (Beckman Coulter) for reverse transcription, Q-PCR and/or Northern blot assay. Reverse transcription was carried out following the technical manual of ImProm-ІІ Reverse Transcription System (Promega). Q-PCR was performed using StepOne Real-time System (Applied Biosystems). Northern blot assays were performed by using the BrightStar BioDetect Kit (Ambion). Probes for Northern blotting were created by using an Ambion BrightStar Psoralen-Biotin Nonisotopic Labeling Kit.

### Primer extension

The promoter region of Φ11 integrase was amplified by PCR with primers pint11-F and pint11-R. The DNA ladder was created using ABI PRISM BigDye Terminators kit with primer pint11-R. 5′-FAM-labelled primer pint11-R-EX was purchased from Sangon (Shanghai, China). For primer extension assay, 50 µg of total RNA of *S. aureus* NCTC8325 from the exponential phase was used. The reverse transcription was performed with pint11-R-EX at 42°C for 1 hour according to the manufacture's instructions of Primer Extension System AMV Reverse Transcriptase (Promega). The product of reverse transcription and DNA ladder were then separated by capillary electrophoresis and fluorescent signals were collected by ABI3770 sequencer (Applied Biosystems).

### Construction of *sigH* and Φ11 *int* mutant strains

The *sigH* and *int* genes of Φ11 in *S. aureus* RN4220, MW2 and/or NCTC8325 were knocked out using a temperature sensitive shuttle vector pMAD [49]. DNA sequences of about 600 bps, located at the up- and down-stream of ORFs of target genes, were amplified by PCR and inserted into pMAD consecutively to obtain pMADΔ*sigH* and pMADΔ*int11*. The constructed plasmids were first transformed into *S. aureus* strain RN4220 and later transferred into MW2 or NCTC8325 by electroporation. Mutant strains were obtained by a two-step screen method as previously described [49]. In the mutants, only the ORFs of target genes were deleted and no extra genes (i.e. antibiotics resistance genes) were introduced into the bacterial genomes.

### *In vitro* transcription

To obtain sigH and sigA proteins for assays *in vitro*, *sigH* and *sigA* genes were amplified from the *S. aureus* NCTC8325 genome by PCR and inserted into pET22b at the site of NdeІ/XholІ to generate pET22b*sigA* and pET22b*sigH.* The plasmids were transformed into the *E. coli* strain Rosetta (DE3) to express his-tag fusion proteins under the induction of 1 mM IPTG at 16°C. Target proteins were purified from cell lysate by Ni-NTA resin, eluted and then dialyzed to remove imidazole.

The promoter regions of *cro* and *int* of Φ11 were amplified with primers pcro11-F, pcro11-R, pint11-F and pint11-R by PCR and purified using QIAquick PCR Purification Kit (Qiagen) to obtain the DNA templates containing *pcro* or *pint*.

One microgram of *E. coli* core RNA polymerase (Epicentre) was pre-incubated with or without sigA or sigH (400 ng) at 37°C for 5 min. Then, 5 µl of DNA template (1 µg), 1 µl of NTP mixture (2.5 mM each), 10 µCi of [α-^32^P]UTP (5000 Ci/mM) and 40 U of RNase inhibitor (Takara) were added to form a final volume of 50 µl containing 0.04 M Tris-Cl (pH 7.5), 0.15 M KCl, 10 mM MgCl_2_, 0.01% Triton X-100 and 0.02 M dithiothreitol. The mixture was incubated at 37°C for 20 minutes, and the reaction was stopped by 0.2 M sodium dodecyl sulfate. The reaction products were purified by phenol-chloroform extraction and then ethanol precipitation. The pellet was resuspended in formamide and denatured at 95°C for 5 minutes. Samples were then separated on an 8% polyacrylamide gel with 6 M urea for radioautography.

### Detection of σ^H^-*pint* recognition *in vivo*

To detect the σ^H^-*pint* recognition *in vivo*, a conditional replication and integration plasmid pAH125 was deployed [29]. The promoter region of Φ11 *int* including SD sequence was amplified with the primers pint11-AH125-F and pint11-AH125-R by PCR. The amplicon was cloned into multiple cloning site of pAH125 at the site of PstІ/EcoRІ to create *pint-lacZ* fusion. Construction of pAH125*pint* was operated in the *E. coli* strain BW25142. Plasmid pAH125*pint* was transformed into the *E. coli* strain ZK126 and then integrated into the host genome with the help of pINT^ts^ to gain strain TW0901 as previously described [29].

The T7 promoter of pET22b*sigH* was replaced by *tac* promoter with *lac* operator from pGEX-2T at BglІІ/NdeІ site to generate pET22b*tac-sigH*. Strain TW0902 was later obtained by introducing pET22b*tac-sigH* into TW0902.

Overnight grown cultures of ZK126, TW0901, and TW0902 were diluted into 50 ml of LB medium, respectively, with an OD_600_ = 0.05 to start the incubation at 37°C (200 rpm). After OD_600_ reached 0.7, the cultures were transferred to 16°C, with or without 1 mM IPTG induction, for an additional 4 hours. The harvest cultures were centrifuged to remove the liquid medium and resuspended in phosphate buffer containing 60 mM Na_2_HPO_4_, 40 mM NaH_2_PO_4_ (pH 7.0), 10 mM KCl, 1 mM MgSO_4_ and 50 mM β-mercaptoethanol to an OD_600_ = 1.0 for lysis. The activity of β-galactosidase was determined as previously described [50].

### Phage induction and lysogenization

Phage induction by UV treatment was performed as previously described [51]. One milliliter of culture at exponential phage (OD_600_ = 0.6) was diluted in 9 ml of chilled phosphate buffered saline (PBS, pH 7.4). The mixture was irradiated with a dose of 50 J/m^2^ on 9 cm diameter Petri dishes. Two milliliters of 5×TBS was immediately added after the irradiation. The culture was then incubated at 37°C in the dark for an additional 2 hours. Phage titers were determined by the traditional double layer method [52] using RN4220 as the indicator strain.

To obtain the phage lysate from lysogens, 2 mg/ml mitomycin-C was added into the cultures at exponential phase, followed by further incubation for 4 hours [53]. Supernatants were sterilized using 0.45 pore diameter membrane filters (Millipore). Phage particles were concentrated by precipitation with polyethylene glycol as previously described [54].

The phage lysate from NCTC8325 by treatment with mitomycin-C was spotted on the susceptible strain RN4220. Survival clones from the center of the plaques were picked and checked for lysogeny by PCR. RN4220Φ11, RN4220Φ12, and RN4220Ф13 were obtained for subsequent experiments by using this method.

To measure the lysogenization ability of the *S. aureus* strains, Φ11 lysate with a titer of about 5×10^8^ was prepared. Susceptible cells were mixed with Φ11 particles with a multiplicity of 50 in PBS containing 10 mM Mg^2+^ at 37°C for 4 hours. The supernatants were then discarded to remove the free viral particles. Pellets were resuspended in PBS and plated onto agar plates. The survival clones were checked for lysogeny by cross-streak assays, which were performed as follows. The bacteriophage lysate was applied as a narrow band across the center of an agar plate, and bacteria were streaked across the dried lysate. After incubation at 37°C for 12 hours, a clear spot was visualized at the cross if the tested bacteria were susceptive cells but not the lysogens.

### List of accession numbers

Genbank accession number of *S. aureus* NCTC8325: NC_007795.

Genbank accession number of *S. aureus* MW2: NC_003923.

Genbank available phage genomes: NC_004615 (Φ11), NC_004616 (Φ12), NC_004617 (Φ13), NC_007047 (Φ187), NC_007051 (Φ2638A), NC_007055 (Φ37), NC_007053 (Φ3A), NC_007052 (Φ42E), NC_007060 (Φ55), NC_007049 (Φ53), NC_007062 (Φ52A), NC_007061 (Φ29), NC_007048 (Φ69), NC_007059 (Φ71), NC_007050 (Φ85), NC_007063 (Φ88), NC_7064 (Φ92), NC_7057 (Φ96), NC_007065 (ΦX2), NC_005356 (Φ77), NC_009526 (Φ80α), NC_007056 (ΦEW), NC_002951 (ΦCOL), NC_003288 (ΦETA), NC_008798 (ΦETA2), NC_008799 (ΦETA3), NC_010147 (ΦMR11), NC_010808 (ΦMR25), NC_004740 (ΦN315), NC_008583(ΦNM1), NC_008617 (ΦNM3), NC_008689 (ΦPVL108), NC_011612 (ΦSauS-IPLA35), NC_011614 (ΦSauS-IPLA88), NC_002661 (ΦSTL), NC_002321 (ΦPVL), NC_002486 (ΦPV83), NC_007058 (ΦROSA), NC_007055 (Φ37), NC_008722 (ΦCNPH82), NC_008723 (ΦPH15), NC_007168 (ΦSH1), NC_007457 (ΦCherry), NC_009815 (ΦA006), NC_001884 (ΦSPBc2), NC_005822 (ΦLC3), NC_005294 (ΦEJ-1), NC_009819 (ΦP9), NC_003524 (Φ3626), NC_009231 (ΦC2).

Genbank GeneID of *S. aureus* NCTC8325 *sigH*: 3920368.

Genbank GeneID of *S. aureus* MW2 *sigH*: 1002599.

Genbank GeneID of *S. epidermidis* RP62A *sigW*: 3241810.

Genbank GeneID of *S. haemolyticus* JCSC1435 *sigH*: 3482668.

Genbank GeneID of *B. anthracis* Sterne *sigH*: 2851339.

Genbank GeneID of *B. subtilis* 168 *sigH*: 936150.

Genbank GeneID of *Clostridium difficile* 630 *sigH*: 4916669.

Genbank GeneID of *Clostridium perfringens* 13 *sigH*: 990784.

Genbank GeneID of *Listeria monocytogenes* Clip81459 *sigH*: 7703665.

Genbank GeneID of *S. aureus* NCTC8325 Φ11 *int*: 1258054.

## Supporting Information

Figure S1 Gene arrangement of staphylococcal prophages. Accessory virulence genes are usually harbored in the lysis region.
(0.17 MB TIF)

Figure S2 Transcriptional analysis of *int* and ORF-C in Φ12 and Φ13. (A) Northern blots containing 5 μg of total RNA of RN4220 (lane 1), RN4220Φ12 (lane 2), RN4220ΔsigHΦ12 (lane 3), and RN4220ΔsigHcΦ12 (lane 4) were probed with Φ12 int probe (left). The membrane was stripped and then reprobed with Φ12 ORF-C probe (right). (B) Northern blots containing 5 μg of total RNA of RN4220 (lane 1), RN4220Φ13 (lane 2), RN4220ΔsigHΦ13 (lane 3), and RN4220ΔsigHcΦ13 (lane 4) were probed with Φ13 *int* probe (left). The membrane was stripped and then reprobed with Φ13 ORF-C probe (right). Ethidium bromide stained 16S rRNA patterns are shown as indications of RNA loading. RL6000 (Takara) RNA marker (lane M) was used to estimate the molecular weight of the fragments.
(2.92 MB TIF)

Figure S3 The primer extension assay was used to determine the 5′ end of the 1.1-kb transcript. (A) The migration position of the extended product is marked by an asterisk. (B) The initial site of the transcription is indicated by an arrow. The SD sequences and the translation start codon are indicated by underline,
(0.72 MB TIF)

Figure S4 SigH mRNA transcription detection by reverse transcriptional PCR. SigH mRNA transcription in WT and ΔsigHc of *S. aureus* NCTC8325 and MW2 was detected by reverse transcriptional PCR analysis, and no PCR product was observed in ΔsigH.
(0.21 MB TIF)

Figure S5 Transcriptional level of *sigH* varies in different growth stage. *S. aureus* MW2 cells were inoculated in 50 ml of TSB medium to start with OD_600_ = 0.05. Cells were collected in lag phase (EE, OD_600_ = 0.2), mid-exponential phase (ME, OD_600_ = 0.6), late-exponential phase (LE, OD_600_ = 2.4), and stationary phase (S, OD_600_ = 4.0), respectively. Extracted RNAs were qualified and quantified by measurement of A_260_/A_280_. Standard deviations are indicated on bars.
(0.20 MB TIF)

Figure S6 Phage excision detection by PCR. (A) Φ11 integrase expression in WT and ΔΦ11intc of *S. aureus* NCTC8325 was detected by reverse transcriptional PCR analysis, and no PCR product was observed in ΔΦ11int. (B) Φ11 *int* mutant caused a permanent lock of Φ11 phage genome on host chromosome, and the excision was recovered in the complementation.
(0.23 MB TIF)

Figure S7 Phage integrase stabilizes the lysogeny in *S. aureus*. (A) The comparison of the transcriptional level of Φ11 *int* in RN4220Φ11 versus the overexpression strain RN4220Φ11+PLI50Φ11int. (B) The comparison of the prophage excision rate between RN4220Φ11 and RN4220Φ11+PLI50Φ11int. (C) The comparison of the spontaneous lysis rate between RN4220Φ11 and RN4220Φ11+PLI50Φ11int.
(0.46 MB TIF)

Table S1 Bacterial strains used in this study.
(0.13 MB PDF)

Table S2 Plasmids used in this study.
(0.12 MB PDF)

Table S3 Oligonucleotides used in this study.
(0.08 MB PDF)
