# Supplementary figures and images for: Alternative Sigma Factor σH Modulates Prophage Integration and Excision in Staphylococcus aureus
Source: PLoS Pathog. 2010 May 13;6(5):e1000888. doi: 10.1371/journal.ppat.1000888 (PMC2869324; doi:10.1371/journal.ppat.1000888)

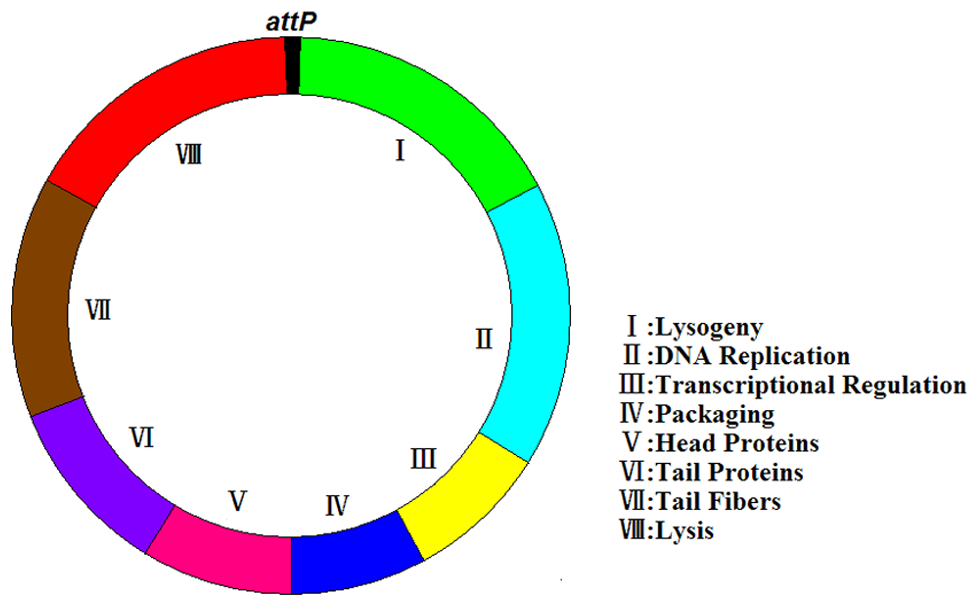

Supplement: Figure S1 — Gene arrangement of staphylococcal prophages. Accessory virulence genes are usually harbored in the lysis region. (0.17 MB TIF) [file ppat.1000888.s001.tif]

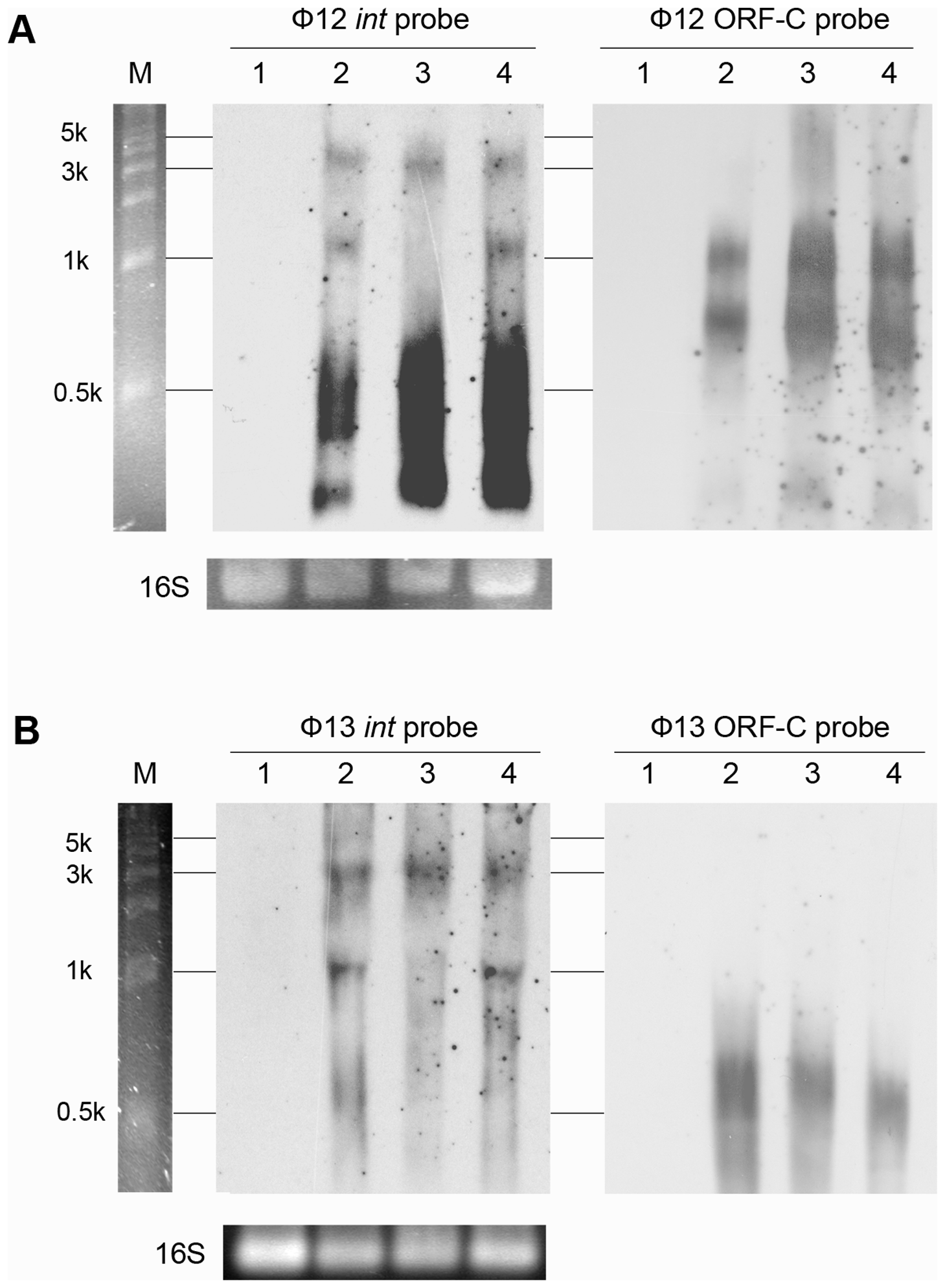

Supplement: Figure S2 — Transcriptional analysis of int and ORF-C in Φ12 and Φ13. (A) Northern blots containing 5 μg of total RNA of RN4220 (lane 1), RN4220Φ12 (lane 2), RN4220ΔsigHΦ12 (lane 3), and RN4220ΔsigHcΦ12 (lane 4) were probed with Φ12 int probe (left). The membrane was stripped and then reprobed with Φ12 ORF-C probe (right). (B) Northern blots containing 5 μg of total RNA of RN4220 (lane 1), RN4220Φ13 (lane 2), RN4220ΔsigHΦ13 (lane 3), and RN4220ΔsigHcΦ13 (lane 4) were probed with Φ13 int probe (left). The membrane was stripped and then reprobed with Φ13 ORF-C probe (right). Ethidium bromide stained 16S rRNA patterns are shown as indications of RNA loading. RL6000 (Takara) RNA marker (lane M) was used to estimate the molecular weight of the fragments. (2.92 MB TIF) [file ppat.1000888.s002.tif]

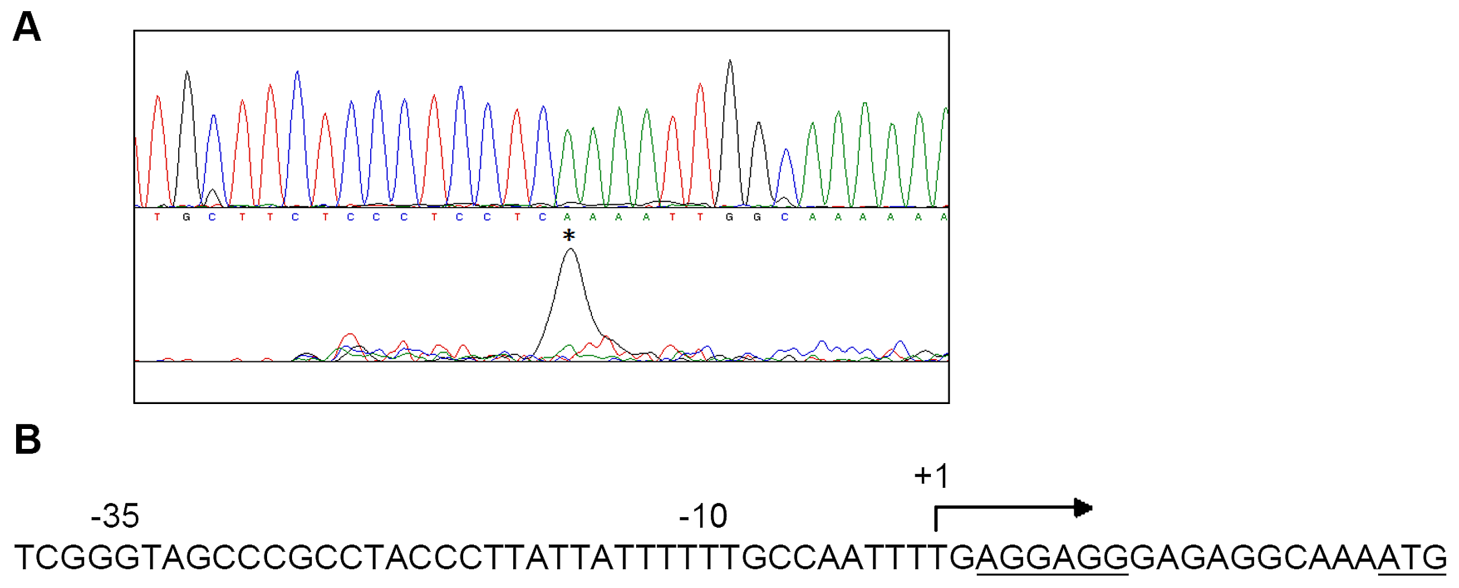

Supplement: Figure S3 — The primer extension assay was used to determine the 5′ end of the 1.1-kb transcript. (A) The migration position of the extended product is marked by an asterisk. (B) The initial site of the transcription is indicated by an arrow. The SD sequences and the translation start codon are indicated by underline, (0.72 MB TIF) [file ppat.1000888.s003.tif]

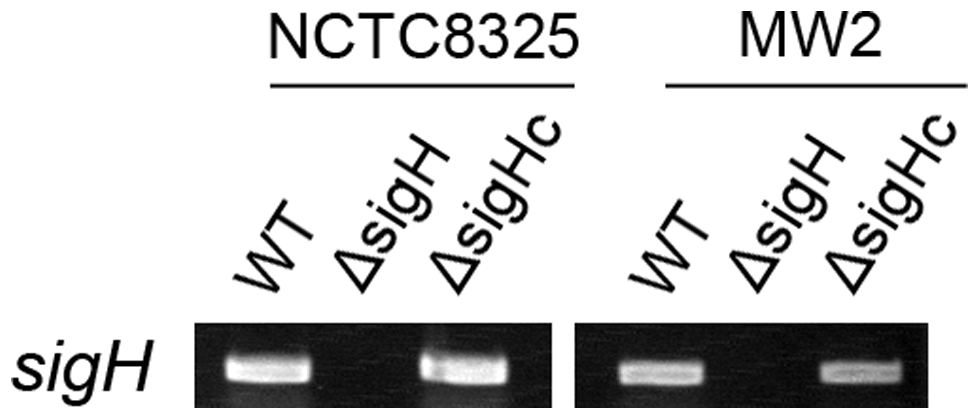

Supplement: Figure S4 — SigH mRNA transcription detection by reverse transcriptional PCR. SigH mRNA transcription in WT and ΔsigHc of S. aureus NCTC8325 and MW2 was detected by reverse transcriptional PCR analysis, and no PCR product was observed in ΔsigH. (0.21 MB TIF) [file ppat.1000888.s004.tif]

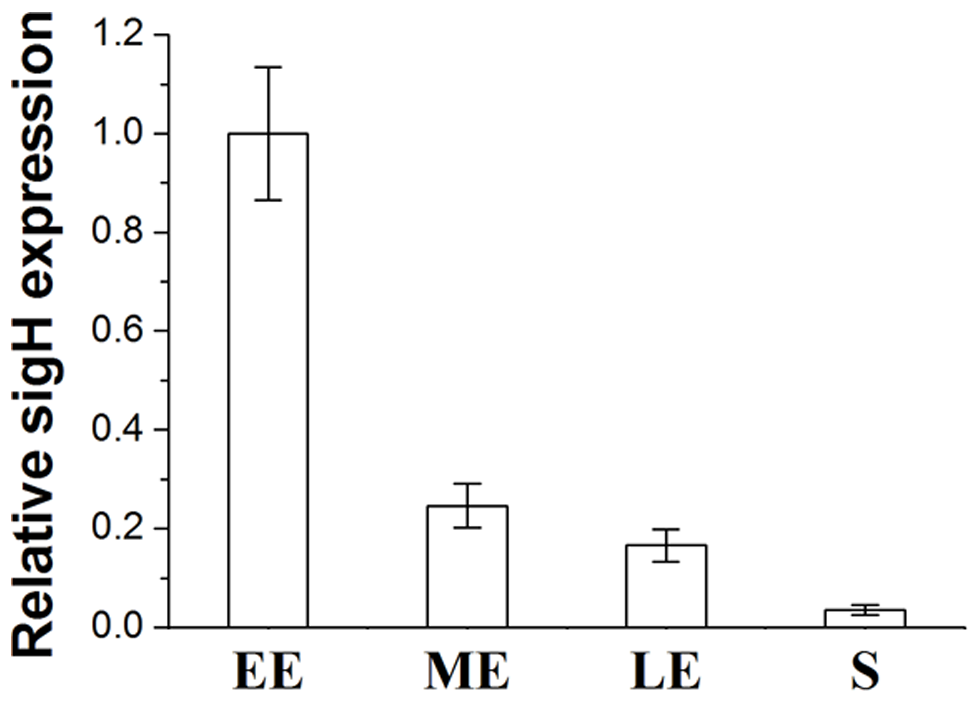

Supplement: Figure S5 — Transcriptional level of sigH varies in different growth stage. S. aureus MW2 cells were inoculated in 50 ml of TSB medium to start with OD600 = 0.05. Cells were collected in lag phase (EE, OD600 = 0.2), mid-exponential phase (ME, OD600 = 0.6), late-exponential phase (LE, OD600 = 2.4), and stationary phase (S, OD600 = 4.0), respectively. Extracted RNAs were qualified and quantified by measurement of A260/A280. Standard deviations are indicated on bars. (0.20 MB TIF) [file ppat.1000888.s005.tif]

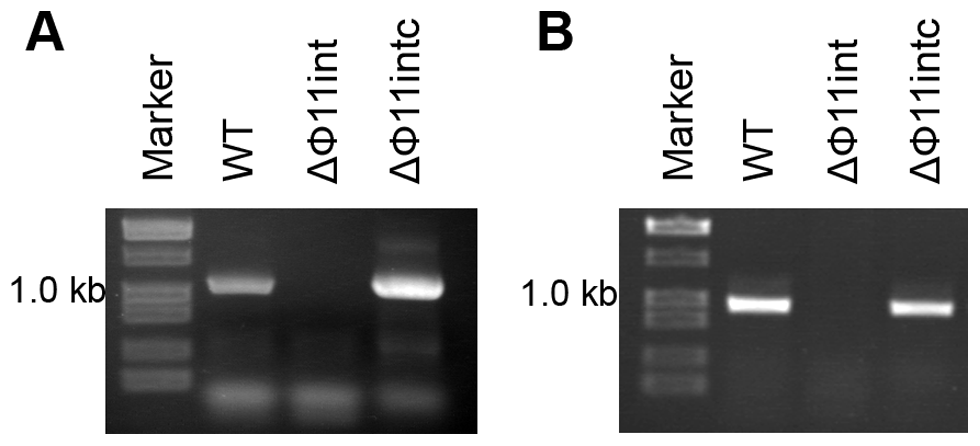

Supplement: Figure S6 — Phage excision detection by PCR. (A) Φ11 integrase expression in WT and ΔΦ11intc of S. aureus NCTC8325 was detected by reverse transcriptional PCR analysis, and no PCR product was observed in ΔΦ11int. (B) Φ11 int mutant caused a permanent lock of Φ11 phage genome on host chromosome, and the excision was recovered in the complementation. (0.23 MB TIF) [file ppat.1000888.s006.tif]

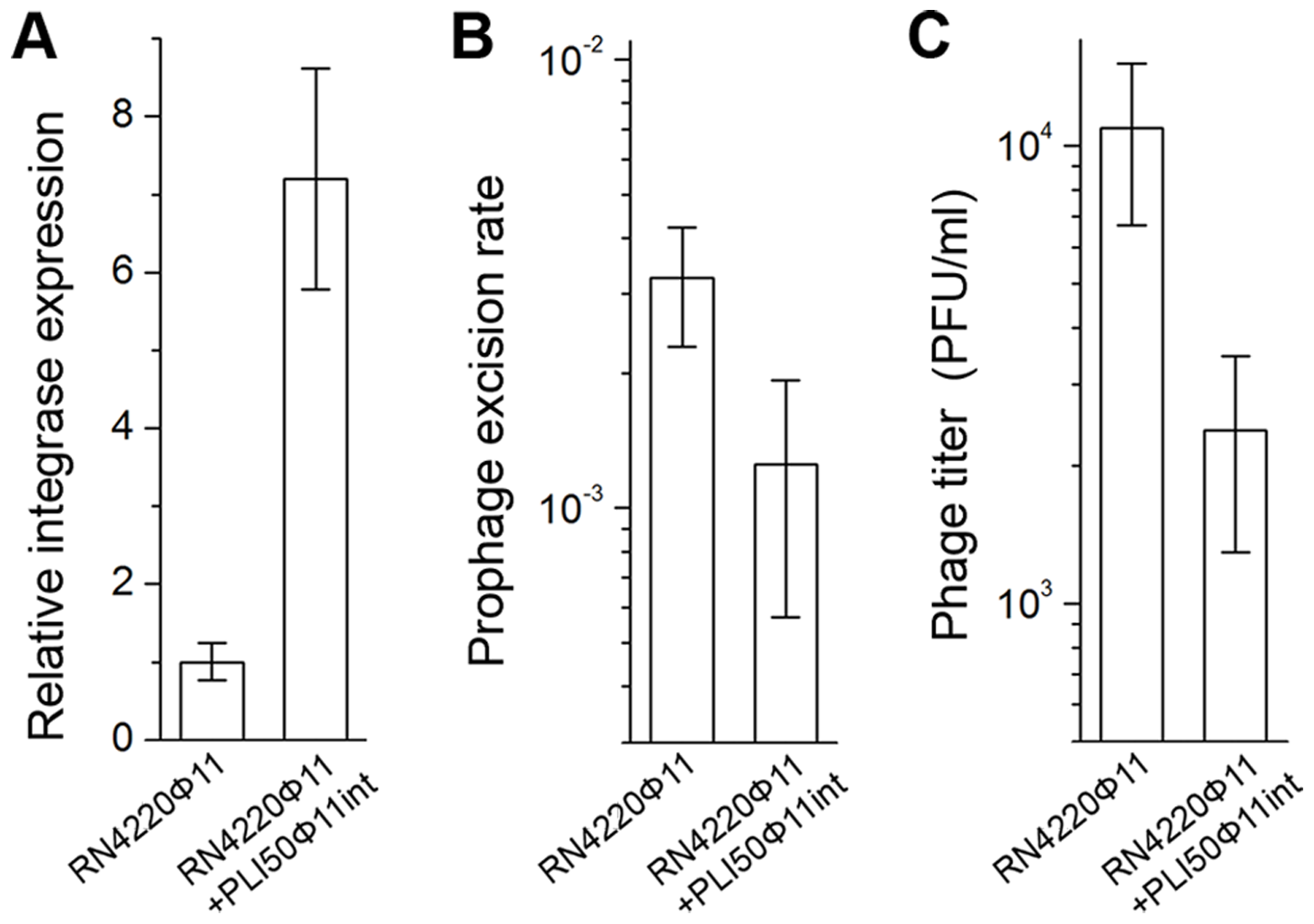

Supplement: Figure S7 — Phage integrase stabilizes the lysogeny in S. aureus. (A) The comparison of the transcriptional level of Φ11 int in RN4220Φ11 versus the overexpression strain RN4220Φ11+PLI50Φ11int. (B) The comparison of the prophage excision rate between RN4220Φ11 and RN4220Φ11+PLI50Φ11int. (C) The comparison of the spontaneous lysis rate between RN4220Φ11 and RN4220Φ11+PLI50Φ11int. (0.46 MB TIF) [file ppat.1000888.s007.tif]
