# Supplementary material for: Alternative Sigma Factor σH Modulates Prophage Integration and Excision in Staphylococcus aureus
Source: PLoS Pathog. 2010 May 13;6(5):e1000888. doi: 10.1371/journal.ppat.1000888 (PMC2869324; doi:10.1371/journal.ppat.1000888)
Supplement: Table S1 — Bacterial strains used in this study. (0.13 MB PDF) [file ppat.1000888.s008.pdf]

Table S1. Bacterial strains used in this study

Species	Strains	Description	Reference or source
E. coli	DH5α	Competent *E. coli* for plasmid transformation	Takara
	Rosetta (DE3)	*E. coli* strain for protein expression	Merck
	BW25142	*lacI*^{q} *rrnB3 ΔlacZ4787 hsdR514* DE(*araBAD*)*567* DE(*rhaBAD*)*568 ΔphoBR580 rph-1 galU95 ΔendA9 uid*A(*ΔMlu*I)::*pir-116 recA1*	[31]
	ZK126	W3110 *ΔlacU169 tna-2*	[32]
	TW0901	ZK126, λ::pAH125*pint*(*pint-lacZ*, Kan^{r})	This study
	TW0902	TW0901, pET22b*tac-sigH*(*sigH*, Ap^{r})	This study
S. aureus	MW2	A community-acquired MRSA strain	[28]
	NCTC8325	Standard laboratory strain	[29]
	RN4220	Derived from NCTC8325-4, selected for transformability with DNA from *E. coli*	[41]
	RN4220Φ11	RN4220 derivative, harboring Φ11	This study
	RN4220Φ12	RN4220 derivative, harboring Φ12	This study
	RN4220Φ13	RN4220 derivative, harboring Φ13	This study
	SUN0802	MW2, *ΔsigH*	This study
	SUN0806	NCTC8325, *ΔsigH*	This study
	SUN0818	NCTC8325, Φ11/*Δint*	This study
	SUN0914	RN4220, *ΔsigH*	This study
