# Supplementary material for: Alternative Sigma Factor σH Modulates Prophage Integration and Excision in Staphylococcus aureus
Source: PLoS Pathog. 2010 May 13;6(5):e1000888. doi: 10.1371/journal.ppat.1000888 (PMC2869324; doi:10.1371/journal.ppat.1000888)
Supplement: Table S2 — Plasmids used in this study. (0.12 MB PDF) [file ppat.1000888.s009.pdf]

Table S2. Plasmids used in this study.

Plasmids	Description[a]	Reference or source
pMD19-T	Commercial TA cloning vector, Ap^r	Takara
pET22b	Commercial vector for protein expression, IPTG inducible, Ap^r	Novagen
pET22b*sigH*	pET22b derivative, harboring *sigH* ORF of NCTC8325, Ap^r	This study
pET22b*sigA*	pET22b derivative, harboring *sigA* ORF of NCTC8325, Ap^r	This study
pGEX-2T	Commercial vector for protein expression, IPTG inducible, Ap^r	GE Healthcare
pET22b*tac*-*sigH*	pET22b*sigH* derivative, T7 promoter is replaced by *tac* promoter, IPTG inducible, Ap^r	This study
$pINT^{ts}$	CRIM helper plasmid, express lambda integrase, Ap^r	[33]
pAH125	CRIM vector, *attP* for chromosomal integration in *attB*-site of lambda phage, Kan^r	[31]
pAH125*pint*	pAH125 derivative, harboring *pint* of Φ11, Kan^r	This study
pMAD	Shuttle vector between *E. coli* and *S. aureus*, Ap^r, Em^r	[42]
pMAD*ΔsigH*	pMAD derivative, containing the sequences up- and downstream of *sigH* ORF, Ap^r, Em^r	This study
pMAD*Δint11*	pMAD derivative, containing the sequences up- and downstream of Φ11 *int* ORF, Ap^r, Em^r	This study
pMAD*sigH*	pMAD derivative, harboring *sigH* ORF and its promoter region of NCTC8325, Ap^r, Em^r	This study
PLI50	Shuttle vector between *E. coli* and *S. aureus*, Ap^r, Cm^r	Addgene, [30]
PLI50*int11*	PLI50 derivative, harboring Φ11 *int* ORF and its promoter region of NCTC8325, Ap^r, Cm^r	This study

[a]Ap^r, ampicillin resistant; Cm^r, chloramphenicol resistant; Em^r, erythromycin resistant; Kan^r, kanamycin resistant.
