# Supplementary material for: Alternative Sigma Factor σH Modulates Prophage Integration and Excision in Staphylococcus aureus
Source: PLoS Pathog. 2010 May 13;6(5):e1000888. doi: 10.1371/journal.ppat.1000888 (PMC2869324; doi:10.1371/journal.ppat.1000888)
Supplement: Table S3 — Oligonucleotides used in this study. (0.08 MB PDF) [file ppat.1000888.s010.pdf]

Table S3. Oligonucleotides used in this study

Name	Sequence (5'→3')
Check11-F	CTAACACATCTTATGTGGC
Check11-R	GTTCTGATAATGTACGTGC
Check12-F	TAAACATAGCTCATCACCC
Check12-R	GAATTTTTGTCCCTGATTCC
Check12-F	TAAAGTTGTTGTAATCAATGAC
Check13-R	GCATATCTTTGAACTCTGG
Check11-IN	CCCGAATCTAAAAATACACC
Check12-IN	CAGCATCAATTCTAGAACCG
Check13-IN	ATTTGCAATTAGCTATGATGG
CheckSa2mw-IN	AGCTATGTTGACAGAGGG
CheckSa3mw-IN	ATATGAAACACATTATGGACG
CI11-F	AGACAGGGTACAGAACTCC
CI11-R	ATCTACACGCTTGATAAGAC
DnaA12-F	TTCGATAGATGGTGGAGCG
DnaA12-R	CTACATCTCGAATACAGTAAT
Amidase13-F	ACACACCATCGTTTGTTCC
Amidase13-R	TCTCATGCCATATCCACTC
RT-SA16S-F	TACGATTACTAGCGATTCCAGC
RT-SA16S-R	TCGAAGCAACGCGAAGAACC
SigH-22b-F	CCACATATGTTGAAATACGATTTGAC
SigH-22b-R	CGACTCGAGAATCATTTTGAAATAACG
SigA-22b-F	CCGAATTCGCGTTTCATGTCTGATAACACAG
SigA-22b-R	CCCTCGAGATCCATAAAGTCTTTCAAACG
Ptac-22b-F	CCCAGATCTACGGTGCACCAATGCTTC
Ptac-22b-R	CCCCATATGGAATACTGTTTCCTGTGTG
SAsigH-UF	CGGATCCTGGATACAATATGATAGGAC
SAsigH-UR	ATATTAAACTAACCCCTTCTATC
SAsigH-DF	AAAGCGCCTTAGGACGTG
SAsigH-DR	CGGATCCCATATCCAGAATATGTATGC
Int11-UF	AACACCTGATTACAAGAGG
Int11-UR	CCACCACCACCACCCATTTACCTGTATTATCATCC
Int11-DF	GGTGGTGGTGGTGGAAAAACATCAAGCTATGAGC
Int11-DR	AGCTTTTAACGGTGGTTGC
SigH-PLI50-F	CGGATCCTGGATACAATATGATAGGAC
SigH-PLI50-R	CAAAAGCTTTCAAATCATTTTGAAATAACG
Pint11-F	GTTTTTAAATGTCATAACATCACC
Pint11-R	GTTCGCTTTTCTATTGAGCG
Pint11-R-EX[a]	GTTCGCTTTTCTATTGAGCG
Pcro-F	ATTCAACAAAAAAATACACG
Pcro-R	CGTCTTCGTTATTCATGC
RT-int11-F	GTTTGAACGTAAGAAAGATGC
RT-int11-R	GTTCGCTTTTCTATTGAGCG
RT-int2mw-F	CTCGGAGATATGAAAGGAGC

RT-int2mw-R	AGTGTCTTTGTATCCGAATCC
RT-int3mw-F	GTTGTTAAAGAAAATACCTACCG
RT-int3mw-R	GTATTTCATATTCAGTTACCGAG
Pint11-AH125-F	AAACTGCAGGGATTCTTCATTACATACCG
Pint11-AH125-R	TTTGAATTCTTTGCTTCTCCCTCCTC
Φ11 *int* probe [b]	GACCTGCATCGTTTAACAAATAATTCCTGAAAGCAGTAC ATTGCGTAGTAGTGATTTTGCC
Φ11 *xis* probe [b]	CAGTAAGCAAAATCGGATTCTTCATTACATACCGAATATT CATCATAAACACTGACTGCATCTTC
Φ11 *cI* probe [b]	CTTCATGCAATATGTCATATAATTCTTCTCCTATGCCAGC ACCAGTTGCACCACATGC
Φ12 *int* probe [b]	GCAGTGCTTGTAGTTCTCCTATGCGCATACCAGTTAAAGC CTGTACTTCTAAGATGCTGG
Φ12 ORF-C probe [b]	ACGGTAAATCAGATACTTTGAAATCTTGTCGCTCAACCTC TAGTAAATCGAAATCGCTACCAGC
Φ13 *int* probe [b]	CGAGTTCTCTTCTTTCTGACGTGATCTTTTAGTATTTCATA TTCAGTTACCGAGATG
Φ13 ORF-C probe [b]	ACGGTAAATCAGATACTTTGAAATCTTGTCGCTCAACCTC TAGTAAATCGAAATCGCTACCAGC

[a]5’-FAM-labelled, HPLC-pure grade

[b]5’-biotin-labelled, HPLC-pure grade
